# Influence of nitrogen functional groups in carbon-based supports anchoring Pt nanoclusters and single atoms for efficient ammonia borane hydrolysis

**DOI:** 10.1039/d5cc06925d

**Published:** 2026-02-05

**Authors:** Ilaria Barlocco, Silvio Bellomi, Bianca M. C. Anghinelli, Xiaowei Chen, Juan J. Delgado, Marta Stucchi, Laura Prati, Karin Föttinger, Alberto Villa

**Affiliations:** a Dipartimento di Chimica, Università degli Studi di Milano via Golgi 19 I-20133 Milano Italy alberto.villa@unimi.it; b Institute of Materials Chemistry, Technische Universität Wien Getreidemarkt 9/165 Wien 1060 Austria; c Departamento de Ciencia de los Materiales, Ingeniería Metalúrgica y Química Inorgánica, Facultad de Ciencias, Universidad de Cádiz Campus Río San Pedro Puerto Real (Cádiz) E-11510 Spain

## Abstract

A Pt(ii) precursor was impregnated onto graphite (C) and carbon nitride (CN_*x*_), successfully forming subnanometric Pt clusters and single atoms. The N species on CN_*x*_ modified the electronic and topological structure of the metal, improving the catalytic properties of the Pt/CN_*x*_ catalyst in evolving hydrogen from NH_3_BH_3_. This was rationalised by combining HR-TEM and XPS characterisation with DFT computational analysis.

## Abstract 2

The N species on CN_*x*_ modified the electronic and topological structure of Pt, improving the catalytic properties of the Pt/CN_*x*_ catalyst in evolving hydrogen from NH_3_BH_3_.

## Graphical abstract

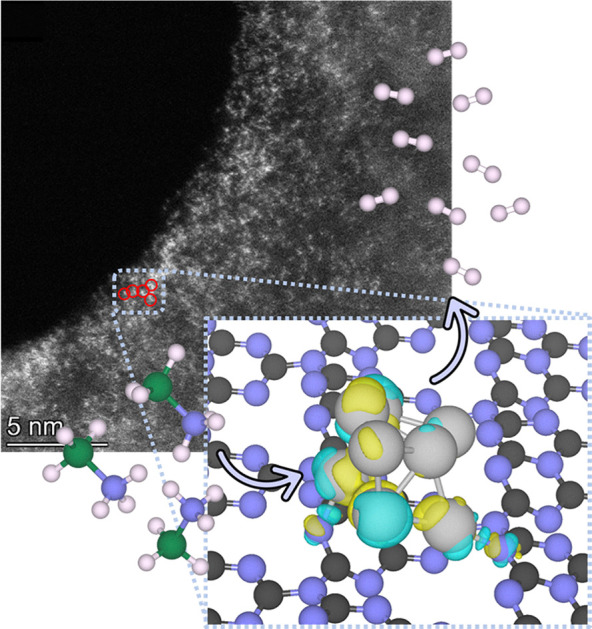

Supported noble-metal nanoparticles (MNPs) have been widely employed as catalysts in various sustainable chemical transformations, such as hydrogen production and biomass valorisation, due to their excellent activity and durability.^1^

It is well recognised that the catalytic properties of MNP-based catalysts are strongly dependent on their physicochemical properties, including structure, composition, shape, and size.^2^ Indeed, the number and coordination of active sites are strictly correlated to the NP diameter, leading to a strong size-activity relationship where “every atom counts”. Usually, the smaller the particle size, the higher the catalytic performance.^3^ In this context, the support plays a fundamental role in tuning the properties of the metal phase. Indeed, MNPs and clusters deposited on different supports may display different reactivity.^4,5^

To this end, carbon materials are very attractive to support a variety of metal species. In fact, they are cheap, and their structure can be easily and finely tuned by introducing defects and heteroatoms.^6,7^ Indeed, various electrocatalytic^8^ and photocatalytic^9^ studies have reported the possibility of obtaining outstanding catalytic properties in hydrogen production reactions by employing N-rich carbon materials as supports for sub-nanometric NPs.

In particular, in our previous work, we have shown exceptional activity, selectivity and stability in hydrogen production from hydrazine hydrate using Ir nanoclusters deposited on graphitic carbon nitride (GCN).^10^ The increased catalytic features were ascribed to the N-containing moieties of GCN, causing electron density redistribution in the supported metal. Hu *et al.* have anchored PtNi NPs on N-doped carbon nanotubes, demonstrating that the N-species accelerate the decomposition of hydrazine to ultra-pure hydrogen and stabilise the metal phase against agglomeration.^10^

Achieving efficient and safe hydrogen production is the key to moving from traditional fossil fuels to “hydrogen energy”.^11,12^ Until now, different hydrogen storage materials have been developed to move this technology forward.

In this context, ammonia borane (AB) is a simple molecular hydride that can be used to produce hydrogen. Its hydrolytic dehydrogenation can be achieved under mild reaction conditions (room temperature and pressure) by means of a heterogeneous catalyst. In this reaction, water is not only the solvent, but also a hydrogen source, see eqn (1). Indeed, 3 moles of hydrogen can be released for one mole of AB.^13^

1 NH_3_BH_3_ + 2H_2_O → NH_4_BO_2_ + 3H_2_(g)

The reaction involves the cleavage of B–H and O–H bonds in AB and H_2_O molecules, respectively. Moreover, a catalyst needs to facilitate the transfer of the hydrogen atoms on the surface to evolve H_2_.^14^ To this end, Pt, Pd and Rh based catalysts were employed to rapidly and selectively decompose AB to molecular hydrogen.^15^ In particular, it was shown that Pt sites can effectively activate and break B–H bonds.^16^ Li and co-workers,^17^ prepared Pt clusters of 1.2 nm embedded in a high surface area Co_3_O_4_ nanocage, and demonstrated that while the reducible oxide support can form H^*δ*+^ from H_2_O, Pt clusters can accelerate the formation of H^*δ*−^ from AB.

It is in this context that this work finds application. Herein, we report the synthesis of Pt sub-nanometric clusters and single atoms supported on graphitic carbon material (graphite and carbon nitride). The Pt(ii) precursor was impregnated on the selected supports and reduced *in situ* by means of the hydrogen produced from AB decomposition. This allowed us to rationalise the catalytic properties of supported Pt on graphitic materials and unveil the role of N-functionalities. Indeed, despite having a similar structure, the materials differ due to the intrinsic N content, profoundly modifying the electronic structure of carbon nitride with respect to graphite, and the subsequent interaction with Pt. To deeply understand the structure–activity relationship and metal–support interaction, the fresh and used catalysts were characterised by HR-TEM and XPS in combination with DFT, giving us an in-depth understanding of the enhanced catalytic properties shown by Pt/CN_*x*_.

Commercial graphite (C) and synthesised carbon nitride (CN_*x*_) were employed as supports for Pt clusters, with a loading of 1 wt%, prepared by the optimisation of the impregnation method proposed by Li and co-workers, and employing K_2_PtCl_4_ as a metal precursor.^18^ The obtained catalysts, Pt/CN_*x*_ and Pt/C, were then employed in the catalytic decomposition of ammonia borane (AB) near room temperature (303 K), with a catalyst : AB molar ratio of 1 : 1000. The pressure of evolved H_2_ was elaborated to obtain the moles of hydrogen *versus* time. The kinetic profiles were evaluated for 100 minutes of reaction, where a plateau indicates the end of the AB decomposition for the most active catalyst, reaching the maximum volume of H_2_ evolved, see Fig. 1. Pt/C was found to be nearly inactive reaching a maximum of 2.102 × 10^−1^ mmol of H_2_ produced, with a turn over frequency (TOF) of 4.23 min^−1^. An evaluation through 300 minutes of reaction did not reveal any change in the shape of the decomposition curve. Notably, passing from graphite to carbon nitride (Pt/CN_*x*_), the moles of hydrogen evolved was found to be 7.455 × 10^−1^ mmol (almost four times more). Moreover, the TOF value increased to 6.36 min^−1^. Because with this set-up the pressure observed is exclusively due to H_2_ produced, we can assess that both catalysts are not capable of completely decomposing AB. It should be noted that the sigmoidal shape observed during the first reaction run in the evolution of H_2_ can indicate the occurrence of structural changes during the reaction. This behaviour can be ascribed to a strong metal support interaction appearing only when Pt and N-functionalities are present. Indeed, in our previous study, we observed a similar trend for AB decomposition on a PtCo_3_O_4_ catalyst, where the induction time was attributed to the transformation of the meta-stable active phase to a modified Pt species, following the Finke–Watzky kinetic model.^20^ On the other hand, during stability tests (Fig. S1), the disappearance of the induction period evidenced the stability of the *in situ* formed active phase. After the fourth run, however, the catalyst showed a decreased initial activity, maintaining the same hydrogen productivity. This could be ascribed to the sintering of Pt small clusters, as confirmed from TEM (Fig. 2j–l).

**Fig. 1:**
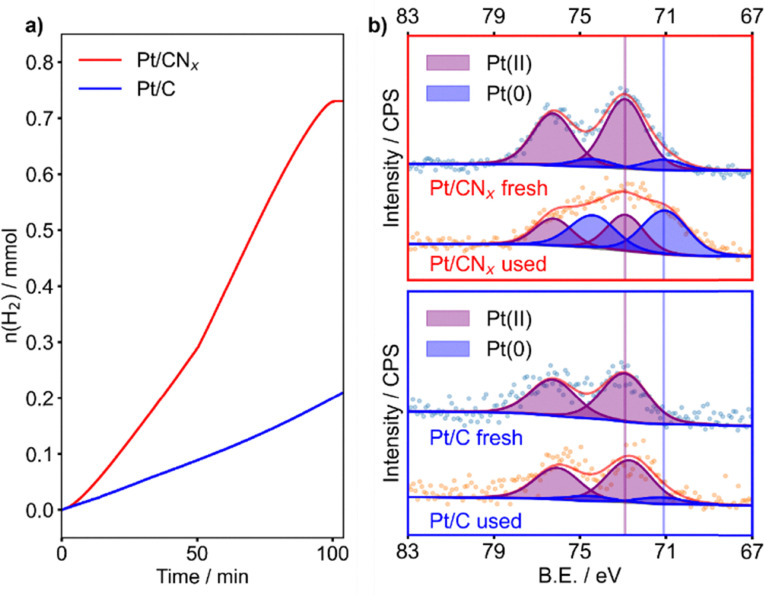
(a) Kinetic profiles of the hydrogen evolution rate of Pt/C (blue) and Pt/CN_*x*_ (red). (b) XPS of Pt/CN_*x*_ (upper panel) and Pt/C (lower panel), fresh and used.

**Fig. 2:**
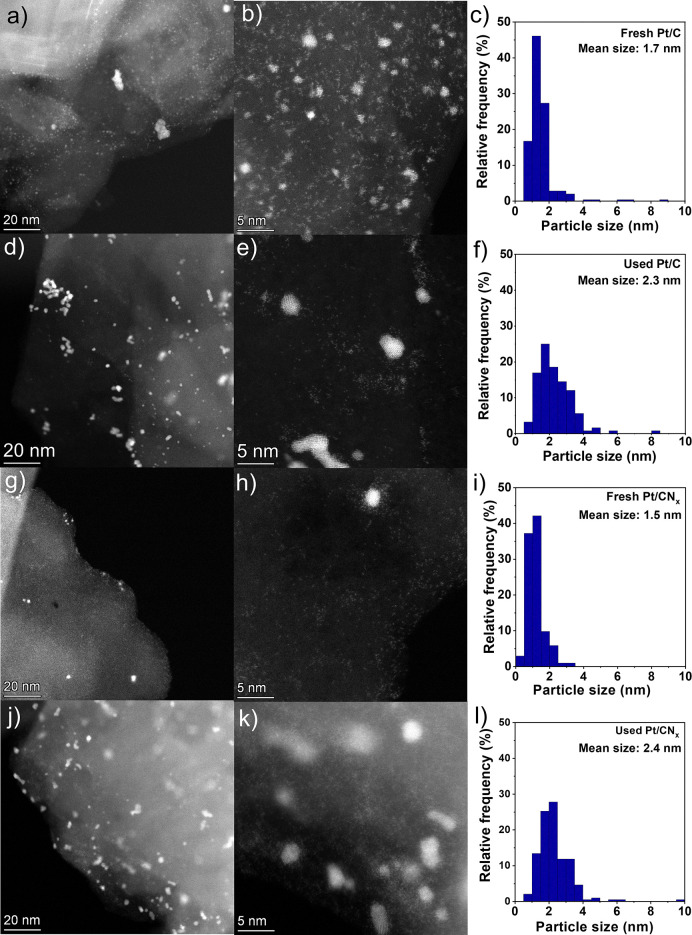
STEM-HAADF images of (a) and (b) fresh Pt/C, (d) and (e) used Pt/C, (g) and (h) fresh Pt/CN_*x*_ and (j) and (k) used Pt/CN_*x*_ catalysts. Particle size distribution of (c) fresh Pt/C, (f) used Pt/C, (i) fresh Pt/CN_*x*_ and (l) used Pt/CN_*x*_ catalysts.

XPS was performed to correlate the information gained by catalysis with the sample surface composition. The results of the survey analysis are summarised in Table S1. We could observe a Pt exposure of only 0.10% for Pt/C, four times less than on Pt/CN_*x*_ (0.39%), despite the similar content of the bulk measured by ICP. The narrow scans of the Pt 4f signals, as well as C 1s and N 1s were fitted using the model described in the SI (Fig. 2b and Fig. S2, S3).

**Fig. 3:**
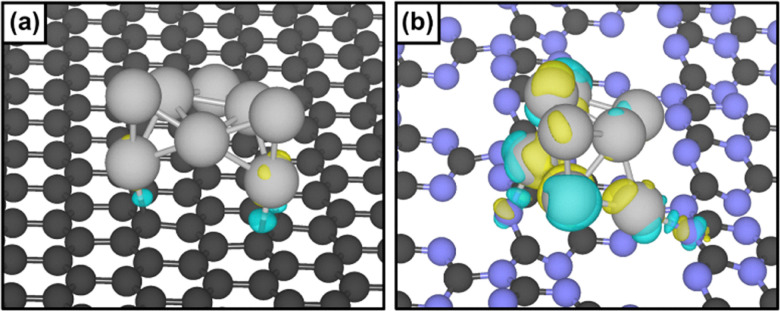
Charge density difference plot of (a) Pt_8_/PG and (b) Pt_8_/CN. Carbon atoms are represented in black, nitrogen in light blue and platinum in grey. Yellow and blue iso-surfaces denote gain and depletion of electron density respectively, and the iso-surface value is 9 × 10^−3^ e^−^ Å^−2^.

The fresh catalysts presented almost uniquely surface Pt^II^: 99.8% for Pt/C and 89.8% for Pt/CN_*x*_, in agreement with the nature of the precursor (K_2_PtCl_6_), and confirmed from the presence of the Cl 2p peak.

On Pt/CN_*x*_, after the reaction, Pt^II^ is reduced to metallic Pt (59.6% of Pt^0^), and the amount of surface Pt remained almost constant (0.35%), but the Cl 2p peak was completely removed. The high content of oxidised species is consistent with a strong electronic interaction with the support.^19^ Indeed, the ratio between the C–N–C and the C=C signal of the CN_*x*_ support decreased from 1.2 to 1.1, and the C–N–C peak shifted negatively by 0.2 eV (Fig. S2 and Table S3), indicating a higher coverage of the N functionalities on the spent catalyst.^19^ This was confirmed by the decrease of the relative percentage of the quaternary N from 8.4 to 7.2% (Fig. S3 and Table S4). On the other hand, in the used Pt/C, Pt was mostly observed as Pt^II^ (13.7% of Pt^0^), although the disappearance of the Cl 2p peak indicated the decomposition of the precursor. The overall amount of Pt decreased (0.06 wt%), suggesting agglomeration of the particles. No significant change was observed from the C 1s signal at the resolution available from the instrument employed. For both catalysts, the lack of any distinguishable B 1s and B KLL peaks was taken as evidence for the absence of residual borates poisoning the active site.^15^

Overall, the XPS analyses corroborate the catalytic test results: the CN_*x*_ support, through its nitrogen functionalities, promotes a strong metal–support interaction that forms and stabilises the Pt active phase during reaction, an effect not observed with the graphite support.

Therefore, the morphology of the fresh materials was investigated with TEM to examine the size and dispersion of Pt clusters on the two supports used: graphite and carbon nitride. Fig. 2 shows representative STEM-HAADF images of the Pt catalysts in their fresh state, as well as the particle size distribution. We could observe that on both the fresh Pt/C and Pt/CN_*x*_, the clusters are homogeneously distributed on the surfaces, indicating a good dispersion. It should be emphasised that it was impossible to measure nanoclusters smaller than 0.5 nm, as shown in Fig. 2b. Excluding nanoparticles smaller than 0.5 nm, the average particle size on Pt/C was 1.7 nm. Most particles fall within the 0.5 to 2.0 nm range, although a few larger particles exceeding 4 nm were observed on the Pt/C catalyst.

However, a few Pt particles on the Pt/C catalyst exceeded 4 nm. In contrast, the particle sizes on the Pt/CN_*x*_ catalyst were all below 3.5 nm and displayed a more uniform distribution. The average particle size was 1.5 nm, without counting very small clusters below 0.5 nm and single atoms (Fig. 2h). This could be attributed to the unique structure of the carbon nitride support, which effectively stabilized both very small clusters and individual metal atoms.^20,21^

The results were corroborated by XRD. For the Pt/CN_*x*_ sample, the diffractograms of bare CN_*x*_ and the catalysts showed the same two peaks, *i.e.* 2*θ* of 13.4° and 27.2° attributed to the (100) and (002) planes of carbon nitride, respectively (Fig. S4).^22^ The absence of Pt-related peaks indicates the presence of small Pt clusters. Comparing the TEM images of both fresh Pt catalysts with the used, an increase of the mean particle size was observed, from 1.7 nm to 2.3 nm for the Pt/C catalyst and from 1.5 nm to 2.4 nm for the Pt/CN_*x*_ catalyst, as shown in the right column of Fig. 2. Although small nanoclusters and single atoms were still present in both the used Pt/C and Pt/CN_*x*_ catalysts, Pt nanoparticles larger than 4 nm were also observed. In addition, the fraction of nanoparticles smaller than 2 nm in both used catalysts is significantly lower than in the fresh catalysts. This could be ascribed to the coalescence of the small clusters during the substrate decomposition to partially form nanoparticles, confirming the transition to another metal phase during the reaction.

To gain a deeper understanding of the metal–support interaction of Pt nanostructures deposited on carbon nitride and to study the enhanced catalytic behaviour of Pt/CN_*x*_ with respect to Pt/C, DFT analysis was performed.

The results of the simulations are presented in Fig. 3 and Table 1. Firstly, pristine graphene (PG) was selected as a model for graphite, and the corrugated carbon nitride structure (CN) characterised by heptazine pores represented CN_*x*_.^23^ Next, an eight atom Pt cluster was optimised on PG and CN (see the SI for the detailed global optimisation procedure). The cluster adsorption on the two different supports was evaluated in terms of adsorption (*E*_ads_), adhesion (*E*_adh_) and deformation (*E*_def_) energies, Table 1, as previously reported.^19^ Indeed, the Pt_8_ cluster was more strongly anchored on carbon nitride than on graphene, demonstrating an enhanced interaction for Pt_8_/CN. Then, an analysis of the charge transfer between the support and the Pt cluster was performed to elucidate its influence on the structure–activity relationship. We observed a net charge redistribution when carbon nitride is the support. In fact, a gain of electron density on the top of the cluster (yellow) and depletion at the interface (blue) was noticed, supporting the superior reactivity due to MSI.

**Table 1:** Energetic and structural information of the Pt_8_ cluster interacting with the supports. Adsorption energy (*E*_ads_), adhesion energy (*E*_adh_), deformation energy (*E*_def_), platinum–carbon distance (*d*Pt–C), platinum–nitrogen distance (*d*Pt–N), and the maximum and the minimum distances between platinum atoms in the cluster (*d*_ma*x*_Pt–Pt and *d*_min_Pt–Pt, respectively) are reported

Structure	*E*_ads_ (eV)	*E*_adh_ (eV)	*E*_def_ (eV)	*d̄*Pt-C (Å)	*d̄*Pt-N (Å)	*d*_ma*x*_ Pt–Pt (Å)	*d*_min_Pt–Pt (Å)
Pt/PG	−2.97	−3.11	−0.14	2.29	—	2.69	2.43
Pt/CN	−3.76	−4.32	−0.56	2.19	2.05	2.66	2.44

In conclusion, in this work, a combination of experimental and computational approaches was employed to elucidate the role of N-functionalities in carbon materials in enhancing the reactivity toward the hydrolytic decomposition of ammonia borane. Two catalysts, Pt/C and Pt/CN_*x*_, were prepared *via* a modified wet impregnation method. The nitrogen species in CN_*x*_ significantly altered the electronic and structural properties of the support, thereby improving the catalytic performance of Pt/CN_*x*_ in hydrogen evolution from ammonia borane.

Interestingly, the Pt/CN_*x*_ catalyst exhibited a sigmoidal hydrogen evolution profile, indicative of a transition from a metastable active species to a more stable one. XPS and TEM analyses revealed that the enhanced activity of Pt on carbon nitride arises from a greater exposure of surface metal species. Moreover, the evolution of the active phase under the reaction conditions was corroborated by particle growth due to coalescence and by partial Pt reduction in the used catalyst.

DFT calculations were performed to model Pt_8_/PG and Pt_8_/CN systems, representing Pt/C and Pt/CN_*x*_, respectively. A stronger interaction between the Pt_8_ cluster and the carbon nitride support was observed, accompanied by charge redistribution within the cluster, accounting for the superior reactivity and metal-support interaction.

The combined experimental and theoretical study highlights that the enhanced catalytic properties of Pt/CN_*x*_ originate from a strong metal–support interaction due to the presence of N-functionalities, providing a coherent explanation for the observed reactivity and characterisation results.

## Conflicts of interest

There are no conflicts to declare.

## Supplementary Material

CC-062-D5CC06925D-s001

## Data Availability

The data supporting this article have been included as part of the supplementary information (SI). The SI file contains the methods (materials, synthesis procedure, catalytic tests, characterisation and DFT modelling) employed in this work, the recycling tests on Pt/CN_*x*_ catalyst and supplementary XPS data. See DOI: https://doi.org/10.1039/d5cc06925d.
