# Supplementary material for: Influence of nitrogen functional groups in carbon-based supports anchoring Pt nanoclusters and single atoms for efficient ammonia borane hydrolysis
Source: Chem Commun (Camb). 2026 Feb 5;62(16):4787–90. doi: 10.1039/d5cc06925d (PMC12895542; doi:10.1039/d5cc06925d)
Supplement: CC-062-D5CC06925D-s001 [file CC-062-D5CC06925D-s001.pdf]

Supplementary Information (SI) for ChemComm.
This journal is © The Royal Society of Chemistry 2026

Supporting Information

Influence of nitrogen functional groups in carbon-based support anchoring Pt nanoclusters and single atoms for efficient ammonia borane hydrolysis

Ilaria Barlocco,[a] Silvio Bellomi,[b] Bianca M. C. Aringhelli,[a] Xiaowei Chen,[c] Juan J. Delgado,[c] Marta Stucchi,[a] Laura Prati,[a] Karin Föttinger[b] and Alberto Villa*[a]

[a]*Dipartimento di Chimica, Università degli Studi di Milano, via Golgi 19, I-20133 Milano, Italy. E-mail: alberto.villa@unimi.it*

[b]*Institute of Materials Chemistry, Vienna University of Technology, Getreidemarkt 9/BC/01, 1060 Vienna (Austria)*

[c]*Departamento de Ciencia de los Materiales, Ingeniería Metalúrgica y Química Inorgánica, Facultad de Ciencias, Universidad de Cádiz, Campus Río San Pedro, Puerto Real (Cádiz) E-11510, Spain*

Supplementary methods

Materials

Graphite was obtained from Johnson Matthey. Pt (II) chloride (H_2PtCl_6, 99.999 %), AB (90%), molten cyanamide, Ludox HS40 SiO_2 particles, NH_4HF_2, were acquired from Sigma-Aldrich.

CN_x synthesis

The carbon nitride sample was prepared following the procedure reported in the literature.[1] Molten cyanamide (1 g, 24 mmol) was heated and stirred at 343 K. Then, SiO_2 particles (2.5 g of a 40 wt% dispersion in water, Ludox HS40) was added dropwise to establish a 40 vol% porosity. The resulting transparent solution was then heated over 2 h at a rate of 4.5 K min^{-1} up to 848 K and then kept at a constant temperature for another 4 h. The obtained yellow precipitate was treated at room temperature over continuous stirring in a closed polypropylene bottle flushed with nitrogen with 25 mL of 4 M NH_4HF_2 for 2 days to remove the silica template. The powder was then centrifuged and washed three times with distilled water and twice with ethanol. Finally, the powder was dried at 343 K under vacuum for several hours.

Pt impregnation

The catalysts were prepared by the optimization of the impregnation method proposed by Li and co-workers.[2] Ideed, the support (200 mg of CN_x and C) was placed in a becker containing 10 mL of H_2O milliQ and the desired amount of metal precursor metal precursor, K_2PtCl_6, was deposited to achieve

a metal loading of 1 wt%. The dispersion was stirred at r.t. for 20 h, filtered, washed with distilled water and dried in oven at 353 K for 4h. The catalysts obtained were labelled Pt/CN_x and Pt/C.

Catalytic tests

Hydrolytic dehydrogenation of ammonia borane (AB) was performed in a 18 mL two-necked round-bottom flask at a constant reaction temperature of 30 °C and a stirring rate of 1400 rpm. Hydrogen evolution was measured by monitoring the pressure of the released gaseous product employing the Man On The Moon X104 kit.[3] Usually, the selected amount of catalyst (Pt/AB molar ratio 1/1000) was added to 5 mL of deionised water and heated to the selected temperature. After equilibrium was reached, 0.4 mmol of AB were injected at once starting the reaction. The kinetic profiles were collected taking 0.5 point s^{-1} until reaction completion, which was indicated by a pressure plateau. The set-up was equipped with an HCl trap capable of capturing NH_3 Under these conditions, the measured pressure is exclusively attributable to the presence of H_2; therefore, the observed conversion of AB directly corresponds to the yield of H_2 produced.

All the tests were performed three times to ensure experimental reproducibility and assess measurement uncertainty.

The supports employed (C and CN_x) were tested under the same reaction conditions and no activity was showed. Blank tests were also performed and AB hydrolytic decomposition results to be significant only at temperatures closed to 80 °C, which are far from the conditions employed in this work.

Turn Over Frequency (TOF) was calculated by considering the slope of the initial linear part of the kinetic curve, normalized for the total moles of Pt in the reaction environment, according to Eq. 1:

$$TOF = n(H_2) * n(Pt)^{-1} * t^{-1} \qquad \text{Eq. 1}$$

Where, n(H_2) are the moles of H_2 produced, n(Pt) the total moles of Pt, t is the reaction time.

Catalyst characterisation

Transmission electron microscopy (TEM) was carried out on a double Cs aberration-corrected FEI Titan3 Themis 60–300 microscope, featuring a monochromator and an X-FEG gun. High-resolution scanning TEM (HR-STEM) imaging was performed at an accelerating voltage of 200 kV, utilizing a high-angle annular dark-field (HAADF) detector with a camera length of 11.5 cm. STEM-HAADF is highly sensitive to the atomic number of elements, with signal intensity approximately proportional to the square of the atomic number (Z^2). This makes it possible to distinguish small Pt nanoparticles supported on lightweight materials such as graphite and CN_x. Using STEM-HAADF images of the catalysts, the diameters of over 100 randomly selected metal particles were measured with ImageJ software, and the corresponding particle size distributions were determined. Based on the particle size

distribution, the mean particle diameter (d) was calculated using the following expression: d=$\Sigma n_i d_i / \Sigma n_i$, where n_i represents the number of particles with diameter d_i.

X-ray photoelectron spectroscopy (XPS) measurements were performed with a SPECS apparatus equipped with an u-Focus 350 Al K_{α} source and a Phoibos 150 WAL detector. For all the source was operated at an emission current of 7 mA and a 14 kV voltage. The materials were grounded to the instrument by means of carbon tape. The data evaluation and fitting were performed using the CasaXPS software. Prior to any analysis, the experimental data were calibrated using the C 1s peak (284.8 eV). Scofield sensitivity factors and a Shirley background type were used for the fitting and quantification of the surface content.

DFT modelling

Spin-polarized periodic plane-wave DFT calculations were carried out using the Vienna Ab-initio Simulation Package (VASP).[4,5] The calculations were performers using projected augmented wave potentials with PBE functionals.[6,7] The following valence electrons were treated explicitly: H (1s), C (2s,2p), N (2s, 2p), O (2s, 2p), Pt (6s, 5d). They have been expanded on a set of plane waves with a kinetic energy cutoff of 400 eV, whereas the core electrons were treated with the projector augmented wave approach (PAW).[6,8] Dispersion forces have been included by the Grimme's D3 parameterization.[9] The threshold criteria for electronic and ionic loops were set to 10^{-5} eV and 10^{-2} eV/Å, respectively. The supports were modelled by considering graphene and corrugated CN_x nanosheet with heptazine pores.[10,11] The optimised supercell parameters are a = 19.749 Å, b = 19.749 Å and γ = 120 °, for graphene and a = 27.693 Å, b = 20.769 Å and γ = 120 °, for carbon nitride. To avoid any interaction between adjacent images, a vacuum > 20 Å was added along the c-axis. The Brillouin zone was sampled using a 3 × 3 × 1 Γ-centred k-point mesh generated through the Monkhorst–Pack method, minimising any Pulay stress.[12]

A metal-cluster model was constructed by considering 8 Pt atoms and placed on the selected supports. For each of the surface slabs, an unbiased genetic algorithm (GA) generated about 200 structures, providing a putative global minimum of the clusters supported, similarly to our previous works.[13] Briefly, the GA involved a pool of 10 members with crossover and mutation operations procedures within a randomization and displacement operators framework. The initial pool members were randomly generated with a cluster-to-surface height of 2.0 Å. A mutation rate of 10% was employed to guarantee the generational variety among the structures. The energy of all these structures was determined using a soft optimization protocol in VASP, i.e., thresholds for electronic and ionic relaxation energies, respectively, of 10^{-4} eV and 10^{-3} eV, evaluated at the Γ-point and with frozen supports. The Brillouin-zone evaluation was eased using the Gaussian method with a smearing width of 0.05 eV.[14] The final Pt_8 structures were then re-optimized at an electronic and ionic relaxation

forces threshold of 10^{-5} eV and 0.02 eV/Å, using a k-points grid of 3 × 3 × 1, including the relaxation of the support.

Adsorption (E_{ads}) and adhesion (E_{adh}) energies where calculated in agreement with Eq. 2 and 3. Here, E_{tot} is the energy of the relaxed final system, E_C the energy of the optimised cluster and E_{Sup} and E_{Sup*} refer to the energy of the relaxed pristine surface and the deformed surface with the geometry of the final system, respectively. In this way, the deformation energy contribution is considered only in the E_{adh} and can be quantified through Eq. 4.

$$E_{ads} = E_{tot} - (E_{Sup} + E_C) \qquad \text{Eq. 2}$$

$$E_{adh} = E_{tot} - (E_{Sup*} + E_C) \qquad \text{Eq. 3}$$

$$E_{def} = E_{ads} - E_{adh} \qquad \text{Eq. 4}$$

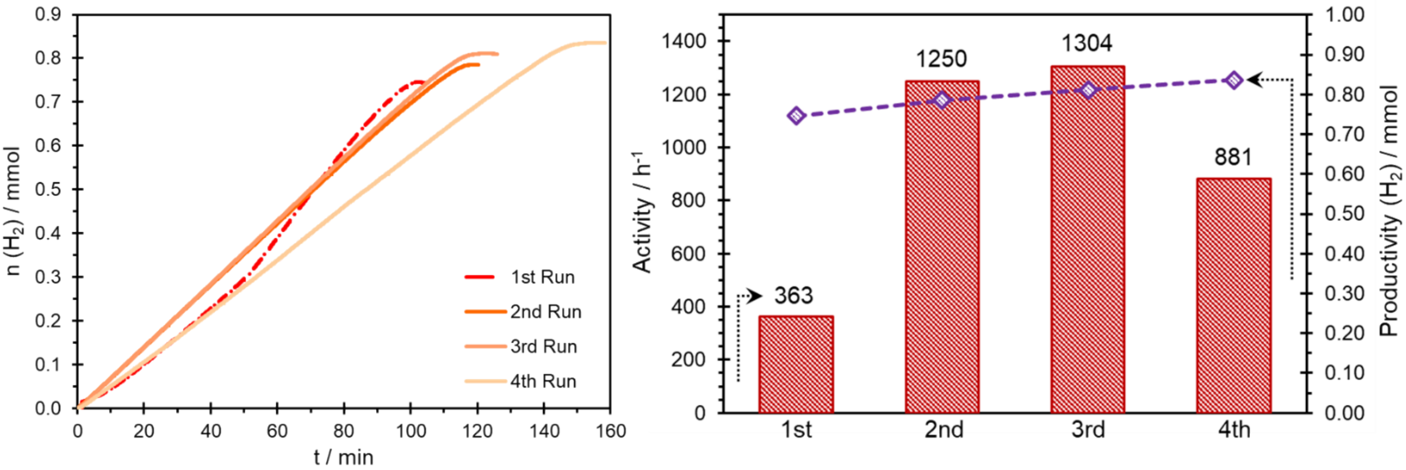

Supplementary figures

Figure S1: Recycling tests of Pt/CN_x catalyst.

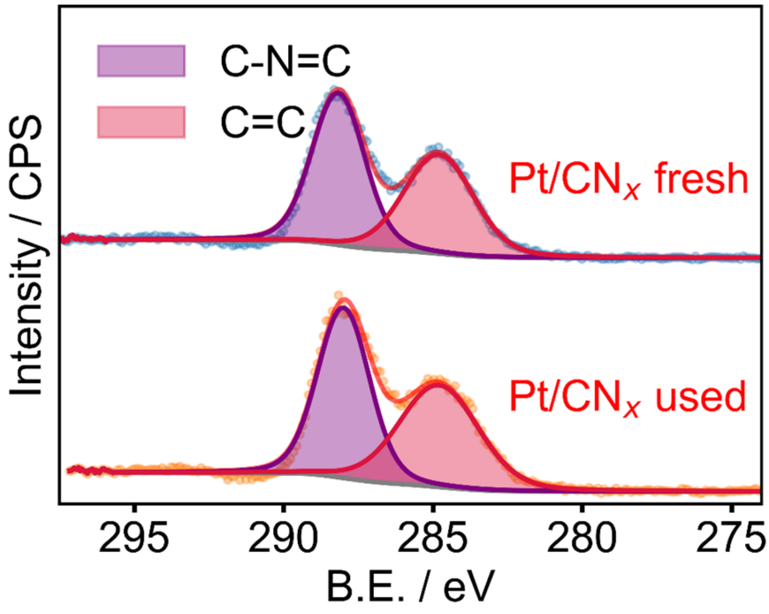

Figure S2: C 1s HR spectra of fresh (upper) and used (lower) Pt/CN_x.

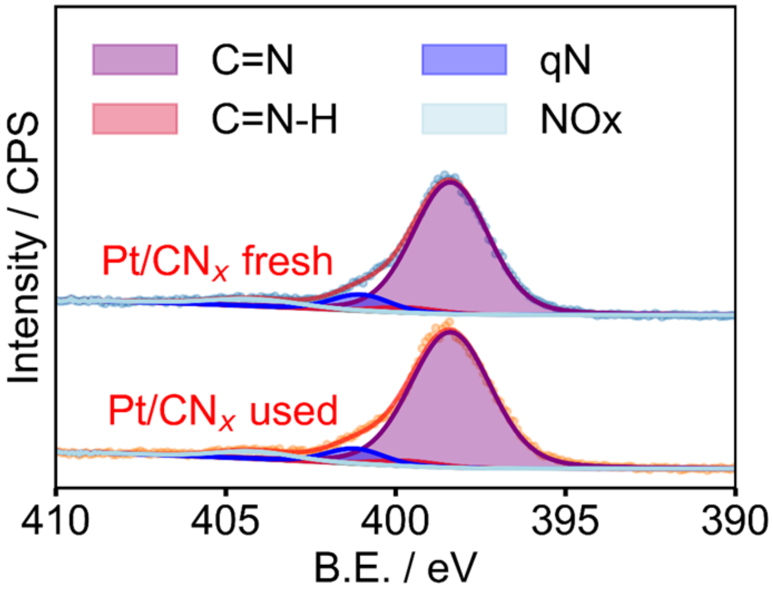

Figure S3: N 1s HR spectra of fresh (upper) and used (lower) Pt/CN_x.

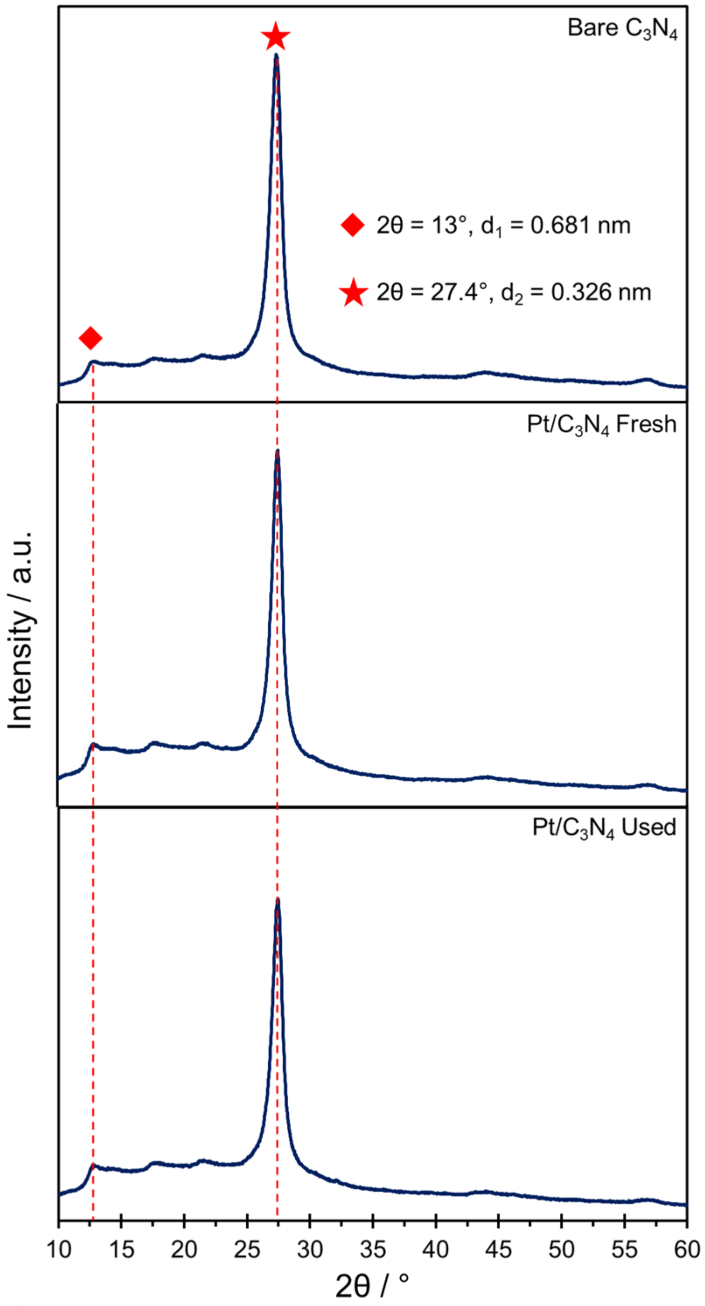

Figure S4: XRD diffractograms of bare and Pt-modified CN_x.

Supplementary Tables

Table S1: Results of survey spectra of fresh and used Pt catalysts.

Sample	Pt 4f / at.%	Cl 2p / at.%	C 1s / at.%	N 1s / at.%	O 1s / at.%
Pt/C fresh	0.10	0.10	97.04	0.00	2.78
Pt/C used	0.06	0.00	97.07	0.25	2.62
Pt/CN_x fresh	0.39	0.47	53.82	40.62	4.69
Pt/ CN_x used	0.35	0.00	52.98	40.98	5.71

Table S2: Results of HR Pt 4f spectra of fresh and used Pt catalysts.

Sample	Pt0 / eV (at.%)	PtII / eV (at.%)
Pt/C fresh	71.0 (0.2)	72.9 (99.8)
Pt/C used	71.2 (13.7)	72.7 (86.3)
Pt/CN_x fresh	71.1 (10.2)	72.9 (89.8)
Pt/CN_x used	71.0 (59.6)	72.7 (40.4)

Table S3: Results of HR C 1s spectra of fresh and used Pt/CN_x catalysts.

Sample	C=N-C / eV (at.%)	C=C / eV (at.%)
Pt/CN_x fresh	288.2 (53.7)	284.8 (46.3)
Pt/CN_x used	288.0 (52.3)	284.8 (47.7)

Table S4: Results of HR N 1s spectra of fresh and used Pt/CN_x catalysts.

Sample	C=N / eV (at.%)	C-N-H / eV (at.%)	qN / eV (at.%)	NO_x / eV (at.%)
Pt/CN_x fresh	398.4 (83.8)	399.7 (2.8)	401.0 (7.9)	404.2 (5.5)
Pt/CN_x used	398.4 (85.8)	399.7 (2.6)	401.2 (6.9)	404.1 (4.7)

Notes and references

1 F. Goettmann, A. Fischer, M. Antonietti and A. Thomas, *Chem. Commun.*, 2006, 4530–4532.

2 Y.-T. Li, X.-L. Zhang, Z.-K. Peng, P. Liu and X.-C. Zheng, *ACS Sustain Chem Eng*, 2020, 8, 8458–8468.

3 S. Bellomi, D. C. Cano-Blanco, Y. Han, J. J. Delgado, X. Chen, K. A. Lomachenko, I. Barlocco, D. Ferri, A. Roldan and A. Villa, *Appl Surf Sci*, 2025, 711, 164116.

4 G. Kresse and J. Furthmüller, *Phys Rev B Condens Matter Mater Phys*, 1996, 54, 11169–11186.

5 G. Kresse and J. Hafner, *Phys Rev B*, 1993, 47, 558–561.

6 G. Kresse and D. Joubert, *Phys Rev B*, 1999, 59, 1758–1775.

7 J. P. Perdew, K. Burke and M. Ernzerhof, *Phys Rev Lett*, 1996, 77, 3865–3868.

8 P. E. Blöchl, *Phys Rev B*, 1994, 50, 17953–17979.

9 S. Grimme, J. Antony, S. Ehrlich and H. Krieg, *Journal of Chemical Physics*, DOI:10.1063/1.3382344.

10 C. Saetta, G. Di Liberto and G. Pacchioni, *Top Catal*, 2023, 66, 1120–1128.

11 S. Bellomi, I. Barlocco, X. Chen, J. J. Delgado, R. Arrigo, N. Dimitratos, A. Roldan and A. Villa, *Physical Chemistry Chemical Physics*, 2023, 25, 1081–1095.

12 H. J. Monkhorst and J. D. Pack, *Phys Rev B*, 1976, 13, 5188–5192.

13 S. Bellomi, D. C. Cano-Blanco, I. Barlocco, J. J. Delgado, X. Chen, L. Prati, D. Ferri, N. Dimitratos, A. Roldan and A. Villa, *ACS Appl Mater Interfaces*, 2024, 16, 54897–54906.

14 J. D. Pack and H. J. Monkhorst, *Phys Rev B*, 1977, 16, 1748–1749.
